# The effect of neo-adjuvant chemotherapy with 5-fluorouracil on the integrity of colonic anastomoses: a systematic review and meta-analysis of experimental studies on rats

**DOI:** 10.3389/fsurg.2026.1737152

**Published:** 2026-03-05

**Authors:** Tatiana Chernyshenko, Roman Polkin, Maksat Ashyrov, Valeriy Shepelev, Roman Goncharuk

**Affiliations:** 1 Department of Clinical Medicine, Far Eastern Federal University, Vladivostok, Russia; 2 Medical Center, Far Eastern Federal University, Vladivostok, Russia; 3 Department of Neurosurgery, 1477th Naval Clinical Hospital, Vladivostok, Russia

**Keywords:** 5-fluorouracil, intestinal anastomoses, meta-analysis, neoadjuvant chemotherapy, systematic review

## Abstract

**Study design:**

Systematic review and update meta-analysis.

**Purpose:**

This systematic review and meta-analysis was conducted to determine the effect of preoperative (neoadjuvant) chemotherapy with 5-fluorouracil (intraperitoneal) on the integrity of colorectal anastomoses in an experimental rat model.

**Overview of literature:**

Emergency surgery in cancer patients undergoing chemotherapy is associated with an increased risk of complications, including anastomotic leakage, and worse survival outcomes. 5FU is widely used in the treatment of GI tumors. The effect of 5FU on anastomotic integrity has been demonstrated previously; this meta-analysis provides a quantitative assessment of this effect.

**Methods:**

A literature search was conducted using PubMed and Google Scholar in MEDLINE up to May 2025. Only experimental studies on rat models were selected, which compared a Control group (no chemotherapy) and a group receiving intraperitoneal 5FU. The following outcomes were extracted: anastomotic bursting pressure, severity of adhesion formation, and hydroxyproline levels.

**Results:**

Six studies were included in the meta-analysis. Statistically significant results demonstrated the superiority of the Control group over the 5FU group in terms of hydroxyproline levels (*p* < 0.00001). The severity of adhesion formation was higher in the group treated with 5FU (*p* = 0.59). The anastomotic bursting pressure was higher in the Control group (*p* = 0.36).

**Conclusions:**

5FU had a negative impact on the integrity of the anastomotic suture line, as evidenced by lower hydroxyproline levels. Lower bursting pressure and a higher degree of adhesions were found in the 5FU group; however, these results were not statistically significant.

## 1 Introduction

Colorectal cancer is the third most common cancer worldwide (1). Surgical intervention remains the primary treatment method. However, despite advancements in surgical techniques, the use of radical treatment does not improve long-term outcomes for locally advanced forms of colorectal cancer (2). It has been proven that resection of the primary tumor induces growth factor activity, which enhances the growth of metastases (3). Therefore, the use of neoadjuvant chemotherapy, particularly with 5-fluorouracil (5-FU), is widely employed to reduce the risk of local recurrence and distant metastases in locally advanced forms and, consequently, increases survival rates (4).

However, chemotherapy is associated with worse outcomes of surgical interventions for acute pathologies, as it can negatively affect wound healing and anastomotic strength (5), subsequently leading to an increase in the length of hospital stay and higher resource intensity of medical care (6). Since situations requiring emergency surgical intervention during ongoing chemotherapy frequently arise in clinical practice, it is crucial to understand how it affects the healing of intestinal anastomoses and to develop treatment and rehabilitation strategies for patients.

To determine anastomotic strength, we used three key parameters: anastomotic bursting pressure, adhesion rate, and hydroxyproline levels. The latter serves as a specific marker of collagen synthesis, a process critical for stabilizing the triple helix of mature collagen and ensuring its mechanical strength. Thus, hydroxyproline concentration indirectly determines the quality of wound healing and the intensity of collagen formation (29).

This study is the first meta-analysis to assess the effect of 5-fluorouracil on anastomotic strength, which underscores the uniqueness of the work. The relevance of the study lies in the systematization and comprehensive assessment of available data, which allows not only for determining the impact of chemotherapy on surgical outcomes but also for laying the groundwork for further clinical research in this area.

Since the question of minimizing the complications of preoperative chemotherapy remains open (7), the aim of our study was to analyze and synthesize existing publications that investigated the effect of preoperative chemotherapy with 5-fluorouracil (intraperitoneal) on the integrity of colorectal anastomoses using meta-analysis technology in an experimental rat model.

## 2 Materials and methods

This review was conducted in accordance with the guidelines of the Preferred Reporting Items for Systematic reviews and Meta-Analyses (PRISMA) (8) and the Assessment of Multiple Systematic Reviews (AMSTAR). A systematic search was conducted using PubMed and Google Scholar via MEDLINE. A highly sensitive search strategy was employed using the following keywords: fluorouracil AND rat AND wound; 5-fluorouracil AND rat AND intestinal anastomoses; “fluorouracil” AND (“Rats” OR “Rat”) AND (“Anastomosis” OR “Anastomoses”); “fluorouracil” AND (“Rats” OR “Rat”) AND (“Anastomosis” OR “Anastomoses”) NOT “cancer”; “fluorouracil” AND (“Rats” OR “Rat”) AND (“Anastomosis” OR “Anastomoses”) NOT “systematic review”. Irrelevant studies were excluded, and duplicates were removed. Only original articles were selected. Additional references were identified through manual screening of the bibliographies of relevant studies, conference abstracts, and registered clinical trials. The search was limited to English-language publications.

### 2.1 Selection criteria

All articles were selected using the previously specified keywords. Data were screened independently by two authors (X), who examined all relevant titles and abstracts to exclude irrelevant publications. The researchers independently assessed the full reports. Subsequently, each selected article was independently evaluated by the entire author team using the PICOS (Population, Intervention, Comparison, Outcome, Study Design) framework (9) and its corresponding inclusion and exclusion criteria (Table 1).

**Table 1:** PICOS. Inclusion and exclusion criteria.

PICOS	Inclusion criteria	**Exclusion criteria**
Population	Wistar rats	Other animal models
	Artificially induced tumor process	
Intervention	Intraperitoneal administration of 5-fluorouracil prior to surgical intervention	Intraperitoneal administration of 5-fluorouracil after surgery;
		Postoperative intraperitoneal administration of other chemotherapeutic agents;
		Intravenous administration of chemotherapeutic agents
Comparison	Intraperitoneal administration of isotonic NaCl solution (0.9%);	Intraperitoneal administration of other chemotherapeutic agents
	No intraperitoneal therapy	Intravenous administration of isotonic NaCl solution (0.9%)
Outcome	Measurement of anastomotic bursting pressure;	Incomplete information on any of the criteria
	Assessment of intra-abdominal adhesions;	
	Hydroxyproline level	
Study design	Retrospective experimental study in rats	Case reports, systematic reviews, meta-analyses
Publications	Full-text publications in English	Publications in other languages, unpublished studies, protocols, conference proceedings and presentations, abstracts, surgical videos

### 2.2 Data extraction and quality assessment

The two aforementioned authors independently extracted data using standardized forms. From the publications meeting the inclusion criteria, information was obtained on the year of publication, study design, intervention, comparative control, overall survival, mean values (Mean) and standard deviations (SD) or confidence intervals (CI), as well as sample sizes. The methodological quality of the studies was assessed using SYRCLE's Risk of Bias Tool (10) and the ARRIVE guidelines (11).

### 2.3 Outcome assessment

The following parameters were analyzed in the study: (1) intraluminal pressure (bursting pressure), (2) adhesion formation, (3) hydroxyproline level.

### 2.4 Statistical analysis

Data analysis was performed using Review Manager ver. 5.4 (The Nordic Cochrane Center, The Cochrane Collaboration, Copenhagen, Denmark). Standardized mean differences (SMD) and their 95% CIs were used for continuous variables. The degree of heterogeneity was assessed using the *I*^2^ statistic. A fixed-effects model was used in the absence of heterogeneity, and a random-effects model was used if *I*^2^was greater than 40%. A funnel plot was constructed, and Egger's test was performed to assess publication bias. A *p*-value < 0.05 was used to indicate statistical significance. Standard deviations were calculated using the guidelines from the Cochrane Handbook for Systematic Reviews of Interventions (12) and Introduction to Meta-Analysis (13).

## 3 Results

### 3.1 Systematic search results

**Figure 1:**
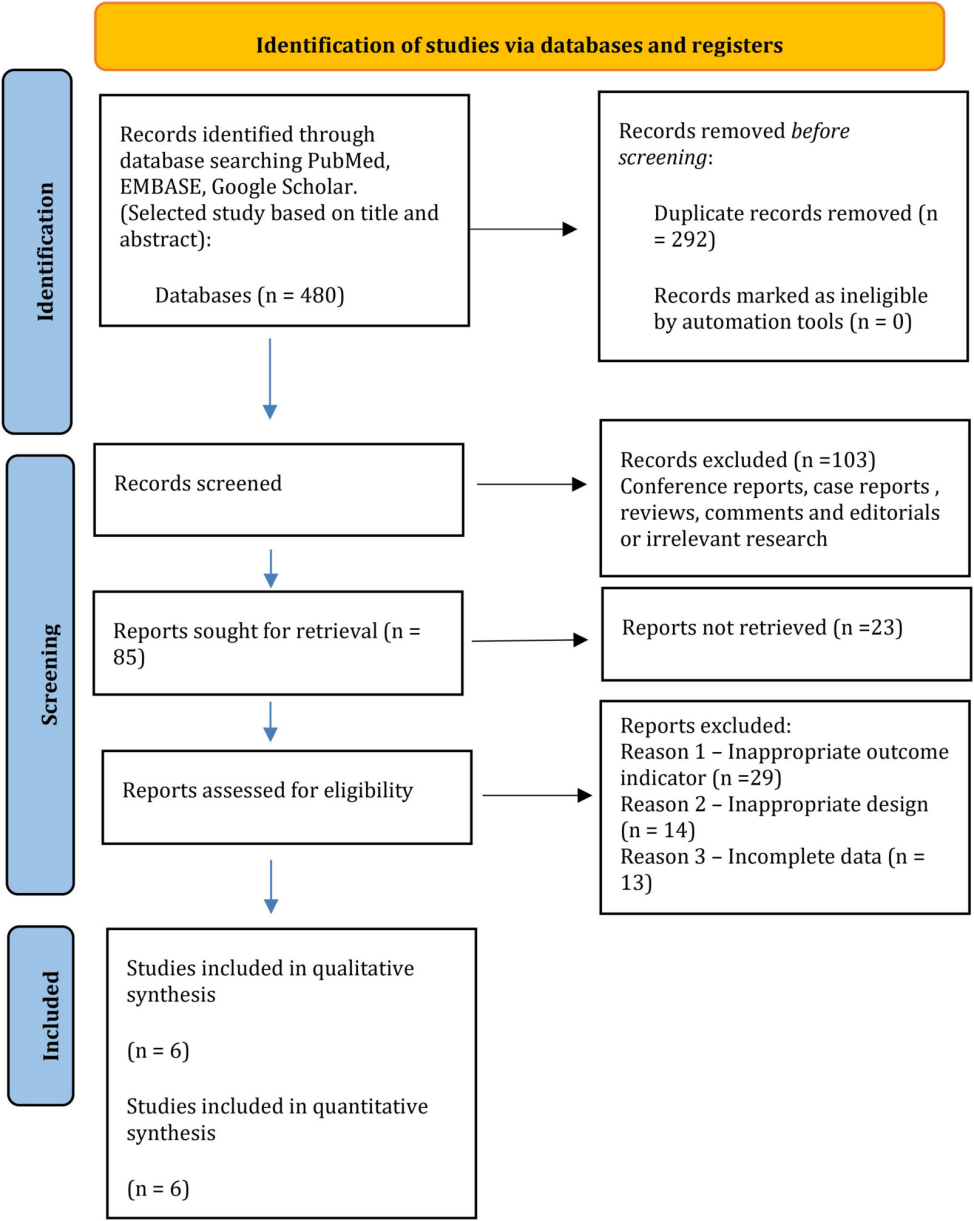
Flow diagram of the studies included in the systematic review according to PRISMA.

Figure 1 provides a summary of the study selection process. A total of 480 articles were identified in the MEDLINE (via PubMed) and Google Scholar databases. In total, 418 studies were excluded as they were duplicates, irrelevant studies, case reports, or reviews. This resulted in 62 potential articles being retrieved for full-text assessment. Of these, 56 articles were excluded for not meeting the inclusion criteria. Finally, 6 studies were included in this meta-analysis (2, 5, 14–17).

### 3.2 Baseline characteristics and quality assessment

Six studies were included in this meta-analysis. Our work did not impose any time restrictions based on publication date and included all relevant works available up to the date of the meta-analysis's publication. The methodological quality of the articles was assessed using two tools: SYRCLE's Risk of Bias Tool (10) and the ARRIVE guidelines (11). The overall quality of the included studies was predominantly low to moderate (Table 2).

**Table 2:** General characteristics of the studies included in the systematic review and meta-analysis. Assessment of ARRIVE scores: A higher score indicates better adherence to international reporting standards in preclinical research.

№	Title	Chemotherapy duration (in days)	Number of Animals		Study Design	5-Fluorouracil dosage (mg/kg) and administration method	Control group	Key Findings	SYRCLE's Risk of Bias Tool	ARRIVE
			5-FU	Сontrol						
1.	El-Malt (5)	10	10	10	Retrospective experimental study in rats	20 intraperitoneal	0.9% saline	In the 5-FU group, more intensive adhesion formation was observed compared to the control group (*p* < 0.01).	Moderate risk of bias	7/10
2.	Kuzu (17)	7	30	20	Retrospective experimental study in rats	20 intraperitoneal	Untreated	No significant differences in anastomotic pressure were found between the groups (*P* > 0.05). Hydroxyproline content was significantly lower after chemotherapy compared to the control group (*P* < 0.01).	High risk of bias	4.5/10
3.	Hananel (14)	7	14	14	Retrospective experimental study in rats	10 intraperitoneal	Untreated	Postoperatively, no significant difference in bursting pressure was observed in the control group. On day 3, rupture occurred at the anastomotic site in all specimens, and on day 7, rupture was observed in the intestinal segment adjacent to the anastomosis in all specimens.	Moderate risk of bias	5/10
4.	Ozel (2)	7	20	20	Retrospective experimental study in rats	20 intraperitoneal	Untreated	Anastomotic bursting pressure values were significantly lower than in the control group (*P* < 0.01). Hydroxyproline levels in the anastomosis were statistically significantly higher in the intraperitoneal administration group compared to the control group. The hydroxyproline level in the intraperitoneal administration group was high (*P* < 0.01).	High risk of bias	6/10
5.	Erenoglu (16)	7	20	20	Retrospective experimental study in rats	20 intraperitoneal	Untreated	The anastomotic bursting pressure in the 5-FU group was lower than in the control group (*p* < 0.05). The hydroxyproline level was significantly higher after chemotherapy (*p* < 0.05).	High risk of bias	4.5/10
6.	Sahin (15)	11	12	12	Retrospective experimental study in rats	20 intraperitoneal	Untreated	In the 5-FU group, a significant decrease in hydroxyproline level was observed compared to the control (*P* < 0.05). In the 5-FU group, the bursting pressure in the area of the colorectal anastomosis was significantly reduced (*P* < 0.05).	Moderate risk of bias	6/10

### 3.3 Clinical study

#### 3.3.1 Anastomotic bursting pressure

**Figure 2:**
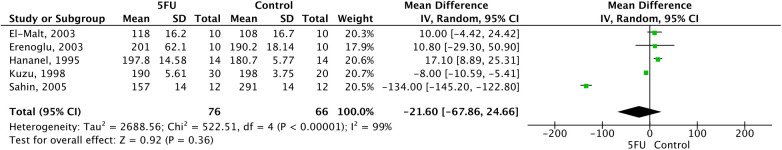
Forest plot of intraluminal pressure.

The control group demonstrated higher values for intraluminal pressure compared to the group receiving 5-FU in the analysis of 5 studies (MD, −21.60; 95% CI, −67.86 to 24.66; *p* = 0.36; *I*^2^ = 99%; random-effects model) (Figure 2).

#### 3.3.2 Intra-abdominal adhesion

**Figure 3:**
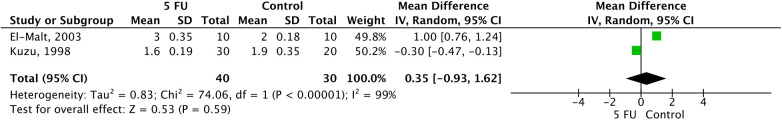
Forest plot of adhesion formation frequency.

According to two studies using the Knightly et al. scale (18), the 5-FU group showed a tendency towards increased adhesion formation compared to the control group (MD, 0.35; 95% CI, −0.93 to 1.62; *p* = 0.59; *I*^2^ = 99%; random-effects model) (Figure 3).

#### 3.3.3 Measurement of hydroxyproline levels

**Figure 4:**
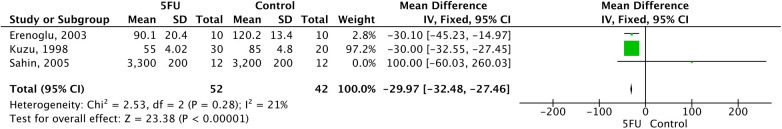
Forest plot of hydroxyproline level.

The analysis yielded a statistically significant result, indicating a higher hydroxyproline level in the Control group (MD, −29.97; 95% CI, −32.48 to −27.46; *p* < 0.00001; *I*^2^ = 21%; fixed-effects model) (Figure 4).

#### 3.3.4 Assessment of publication bias

**Figure 5:**
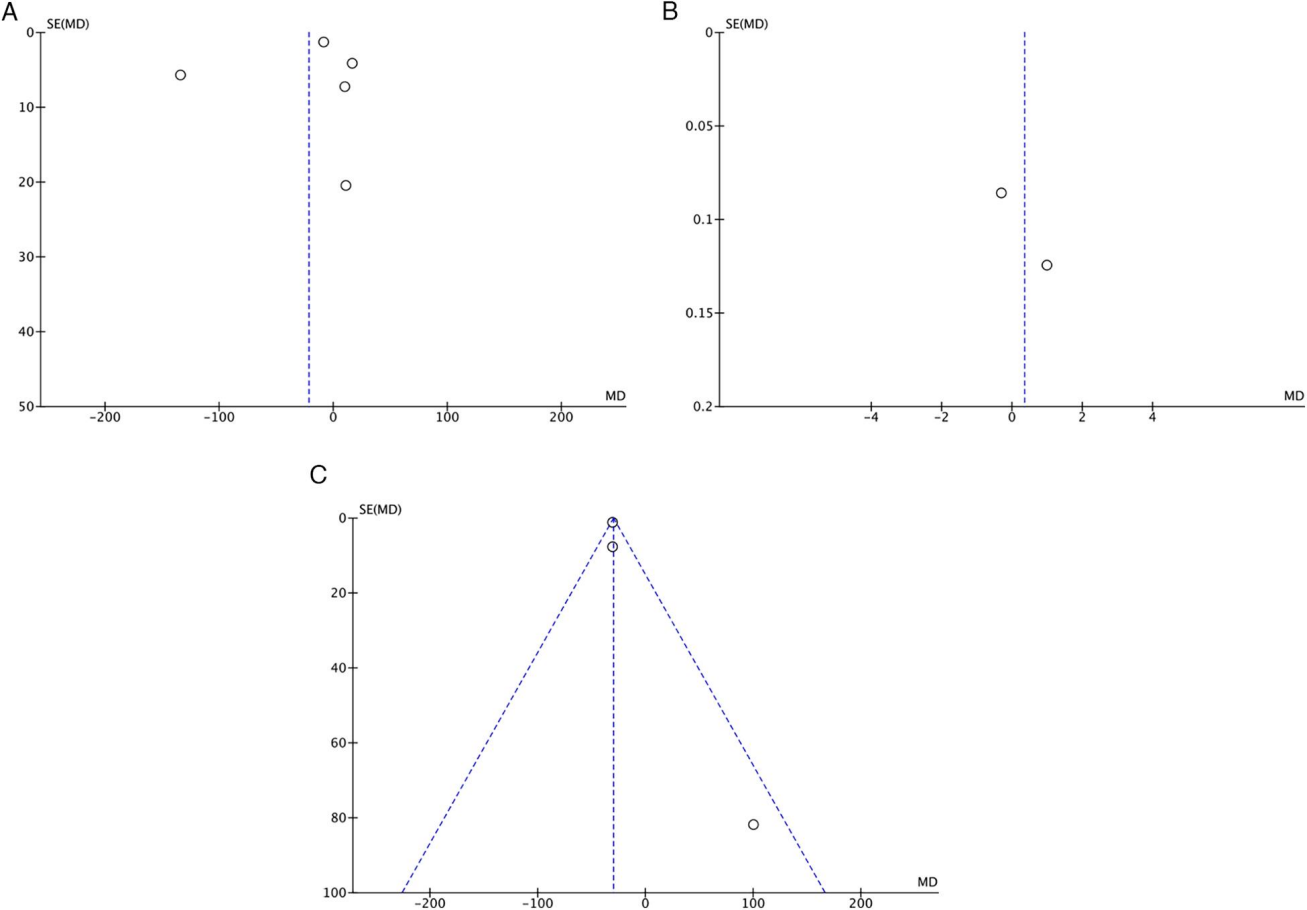
Funnel plots for **(A)** anastomotic bursting pressure. **(B)** Intra-abdominal adhesion. **(C)** Measurement of Hydroxyproline Levels.

Publication bias for each study parameter was assessed through visual inspection of funnel plots. The studies were distributed almost symmetrically on both sides of the vertical line, indicating relatively low publication bias (Figure 5).

## 4 Discussion

In recent years, significant progress has been observed in the comprehensive treatment of malignant neoplasms of the gastrointestinal tract. Neoadjuvant chemotherapy (CT) has proven its effectiveness in treating tumors of various GI locations, including pancreatic, rectal, and gastric cancers (19). According to conducted multicenter randomized clinical trials, neoadjuvant chemotherapy prior to colon surgery led to a significant downstaging in TNM classification compared to the control group (*p* = 0.04), including two pathological complete responses (20, 21). Furthermore, this method is considered a promising direction in the therapy of potentially resectable neoplasms (19). The therapeutic potential of preoperative systemic chemotherapy manifests in several key aspects. First, it contributes to reducing the volume of the primary tumor and increases the possibility of its radical removal (22). Second, it allows for the assessment of the initial response of tumor cells to chemotherapeutic exposure prior to surgical intervention.

The optimal timing for surgical intervention after completing neoadjuvant treatment is strictly regulated by clinical guidelines and varies depending on the location of the tumor process (23, 24). However, in clinical practice, situations often arise that require emergency surgical intervention during ongoing chemotherapy. Under such conditions, the question of assessing the risk of anastomotic leakage during urgent surgical procedures performed while on a chemotherapy course becomes particularly relevant. Despite the existence of clear treatment protocols, this clinical situation requires additional study and the development of an optimal patient management strategy. Addressing this issue could significantly influence treatment outcomes and improve the prognosis for this category of patients.

The mechanisms of anastomotic leakage involve a complex of interconnected processes initiated by impaired microcirculation in the anastomotic zone. Hypoxia directly suppresses the synthesis of collagen, a key structural protein responsible for the mechanical strength of the scar, and creates conditions for the activation of matrix metalloproteinases (MMPs). Concurrently, bacterial contamination exacerbates the destruction of the extracellular matrix through the direct production of bacterial collagenases and stimulation of MMPs, simultaneously triggering an excessive and prolonged local inflammatory response with the release of interleukin-6 and tumor necrosis factor-α. This cascade leads to an imbalance between collagen synthesis and degradation, lysis of developing scar tissue, and, ultimately, to intestinal wall defects (30).

Analysis of our data revealed significant differences between the study groups. Statistically significant results demonstrated the superiority of the Control group over the 5FU group in terms of hydroxyproline levels, indicating a significant impact of the chemotherapeutic agent on anastomotic strength. Assessment of the adhesive process revealed a higher degree of adhesion formation in the group of patients receiving 5FU. The intraluminal pressure level at the anastomosis was higher in the Control group patients. However, these results did not reach statistical significance and require clarification in future studies.

We did not identify any systematic review or meta-analysis that encompassed both clinical and experimental studies on the effect of preoperative 5-fluorouracil (5-FU) use on the healing processes of colorectal anastomoses. The conducted meta-analysis, based on data from six experimental studies using rat models, demonstrated that preoperative use of 5-FU is associated with a significant decrease in hydroxyproline levels at the anastomotic site—a key marker of collagen synthesis—indicating suppressed collagenogenesis. This effect was consistent regardless of the 5-FU administration method or the timing of assessment. Similar data were obtained in studies by Sahin et al. (15) and Kuzu et al. (17), which also noted reduced hydroxyproline content and fibroblast counts in groups receiving 5-FU, correlating with impaired early healing stages. Regarding the mechanical strength of the anastomosis, measured by artificially increasing pressure in the resected anastomosis, our pooled analysis did not reveal a statistically significant difference between the groups (*p* = 0.36) and showed a high heterogeneity coefficient among the studies. Previously, Ozel et al. (2) and Kuzu et al. (17) reported a significant reduction in bursting pressure with intraperitoneal administration of 5-FU 24 h before surgery, while El-Malt et al. (5) found no differences with a longer interval between chemotherapy and surgery. These findings suggest that the time interval between completing chemotherapy and surgical intervention is a critically important factor determining the risk of complications. The degree of adhesion formation showed a tendency to increase in the 5-FU group, with El-Malt et al. (5) reporting a statistically significant increase in adhesions (*p* < 0.01), which may be associated with the intraperitoneal method of drug administration.

Recent clinical studies examining the impact of antitumor drug therapy on outcomes of emergency surgical treatment (21–24) demonstrate that chemotherapy not only disrupts reparative processes at the cellular level but is also associated with a significant increase in the frequency of postoperative complications. For instance, the analysis by Sullivan et al. (25), which included 1,912 patients, showed that emergency surgery performed within 30 days after chemotherapy was associated with a significantly higher rate of serious complications (44.0% vs. 39.2%, *p* = 0.033; OR: 1.20) in the postoperative period, while mortality in this group was more than twice as high compared to the control group (22.4% vs. 10.3%, *p* < 0.001; OR: 2.53) (25). A retrospective analysis by Negruț et al. (26) revealed that emergency surgeries in patients with colorectal cancer are accompanied by a significantly higher frequency of complications, including anastomotic leakage, compared to elective interventions, which is directly related to impaired healing due to chemotherapy (26). Modern clinical observations, such as the study by Ossola et al. (27), demonstrate that patients undergoing emergency surgery during active chemotherapy have a postoperative complication rate of 20%, including infectious complications, anastomotic leakage, and acute peritonitis (27). Even more extensive data are presented in the study by Mallampalli et al. (28), which, based on an analysis of the national NSQIP database, showed that cancer patients who received systemic antitumor therapy within 90 days prior to emergency laparotomy had a significantly increased risk of serious complications (Clavien-Dindo ≥3), infections, and 30-day mortality. Importantly, the rate of anastomotic leakage in this group reached 10.2%, significantly exceeding the rates in both cancer patients without antitumor therapy and non-oncological patients (28).

Thus, the body of contemporary clinical research confirms that emergency surgeries in cancer patients undergoing chemotherapy are associated with an increased risk of complications, including anastomotic leakage, and worse survival outcomes. Our analysis supplements these observations with a quantitative assessment of the pathophysiological mechanisms: we demonstrated that 5-FU significantly reduces hydroxyproline levels—a key marker of collagen synthesis (MD, −29.97 ng/g of tissue; *p* < 0.00001)—which explains the impaired healing. Finally, our work underscores the necessity for a thorough assessment of the patient's condition and a multidisciplinary approach to managing cancer patients requiring emergency surgery.

The conducted meta-analysis has several limitations. First, all included studies are experimental, conducted on animal models, which limits the extrapolation of the results to clinical practice. Second, most studies have a moderate or high risk of systematic bias, including a lack of randomization, blinding in outcome assessment, and adequate description of methods. The average score on the ARRIVE scale was 5.8/10, indicating insufficient adherence to international reporting standards in preclinical research. The high heterogeneity for the primary outcomes (*I*^2^ > 95%) also reduces the reliability of the pooled estimates. Additionally, it is important to note the lack of standardization in chemotherapeutic drug dosages across the analyzed studies, as well as variability in the duration of chemotherapy courses (from 7 to 11 days) and a discrepancy between the drug administration routes used in the studies (predominantly intraperitoneal) and the prevailing practice of intravenous injections in real-world clinical settings. Furthermore, a significant methodological limitation was the restriction of the publication sample to English-language sources only.

## 5 Conclusion

The conducted meta-analysis revealed a significant decrease in hydroxyproline levels following intraperitoneal administration of 5-FU, which led to reduced anastomotic strength and an increased risk of suture line failure. This was further supported by lower intra-abdominal pressure values in the 5-FU group. Although an increase in adhesion formation was observed in the group receiving chemotherapy, these results did not reach statistical significance. The combination of these factors underscores the necessity of conducting clinical studies and further analysis of this patient population to improve outcomes of both surgical and chemotherapeutic treatment.

## Data Availability

The raw data supporting the conclusions of this article will be made available by the authors, without undue reservation.
